# Accidental Ultrasound Finding of a Giant Intermuscular Gluteal Lipoma with Intrapelvic Extension: A Case Report

**DOI:** 10.7759/cureus.7143

**Published:** 2020-02-29

**Authors:** Nikolaos Lazaridis, Nikolaos Anastasopoulos, Alexandros Zevgaridis, Maria Piagkou, Konstantinos Natsis

**Affiliations:** 1 Anatomy and Surgical Anatomy, Aristotle University of Thessaloniki, Thessaloniki, GRC; 2 Anatomy, Aristotle University of Thessaloniki, Thessaloniki, GRC; 3 Surgery, Interbalkan Medical Center, Thessaloniki, GRC; 4 Anatomy and Surgical Anatomy, National and Kapodistrian University of Athens, Athens, GRC

**Keywords:** giant gluteal lipoma, intrapelvic extension, sciatic hernia, pelvic ultrasound

## Abstract

Lipomas represent the most common benign mesenchymal tumor. They are usually located subcutaneously and rarely become symptomatic. Occasionally pressure symptoms on adjacent neurovascular structures may be exerted, thereby causing functional impairment. Lipomas rarely grow larger than 5 cm, becoming the so-called giant lipomas, posing a real diagnostic and surgical challenge. We report an unusual case of a 43-year-old Caucasian female with an accidental pelvic ultrasound finding of a giant mass, which was also palpable over the right gluteal region. Interestingly the patient was free of any sciatic nerve compression symptoms. Magnetic resonance imaging (MRI) revealed a soft tissue tumor, partially located in between the external rotator muscles of the hip and the gluteus muscle. The tumor forced up and advanced through the great sciatic foramen into the pelvis. The patient underwent an uneventful complete and safe surgical excision of the gluteal mass, via a wide transgluteal approach. Pathology confirmed lipoma diagnosis. Patient is free from recurrence two years post operatively. Physicians involved in the diagnosis and treatment of gluteal masses should always consider in their differential diagnosis the presence of a sciatic hernia.

## Introduction

Lipomas are benign mesenchymal tumors. They are the most common type of soft tissue tumor with an annually reported incidence of up to 0.5/1000 [1]. Lipomas are usually found in the subcutaneous tissue and in most cases remain asymptomatic, unless they compress surrounding neurovascular structures. However, deeper located large lipomas, due to their size, could exert pressure symptoms on adjacent nerves and vessels, thereby causing functional impairment [2]. Papen, in 1750, was the first to describe sciatic hernias, which are very rare, while published reports of them scarcely appear in the literature [3]. They are further classified into Type 1 (suprapiriform), Type 2 (subpiriform) and Type 3 (subspinous) being the least common [4]. We report a case of a Type 2 sciatic hernia which was an accidental pelvic ultrasound finding comprising of an asymptomatic giant gluteal lipoma, with an intra pelvic extension through the greater sciatic foramen.

## Case presentation

A 43-year-old Caucasian female, during a regular office visit to her gynecologist underwent an ultrasound scanning and she was accidentally diagnosed with a pelvic mass. Patient was otherwise asymptomatic and well nourished. An easily palpable, reducible lump over the right gluteal area was revealed, consistent with a subcutaneous lipoma. Physical examination did not show any signs of sciatic nerve compression, such as pain radiating down over the buttock, the back of the thigh and calf. Past medical and surgical history was free of any malignancies and traumas in the hip region. No recent weight loss or malaise was reported. On palpation, the abdomen was soft, free of masses or peritonitis signs. Sphincter tone was normal during a digital rectal examination but a soft mass was found in the right ischiorectal fossa.

**Figure 1:**
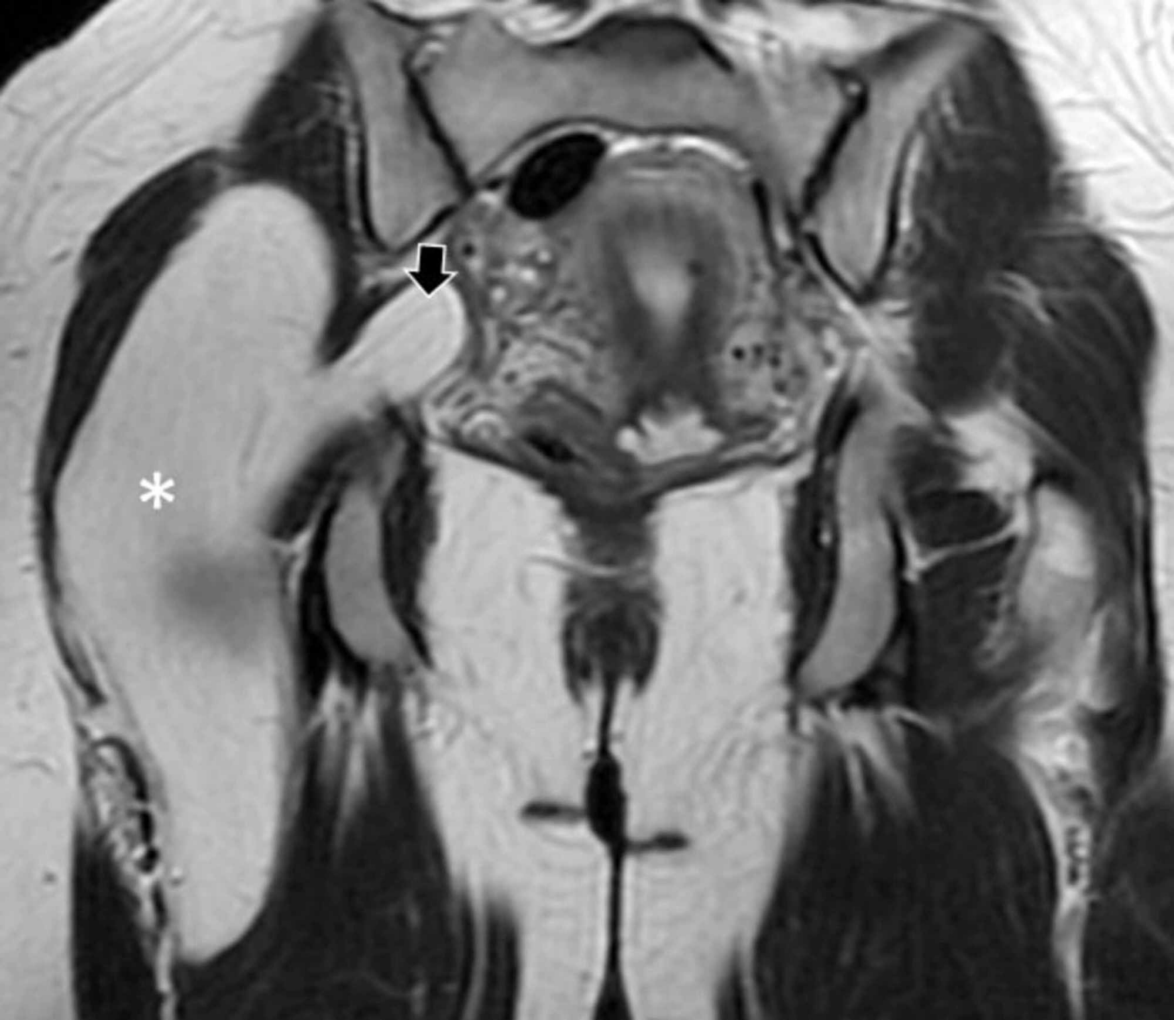
Preoperative magnetic resonance imaging (MRI) of the tumor herniating through the sciatic foramen Coronal T2W image showing an isointense to the fat tissue mass. Arrow indicates the pelvic and asterisk the gluteal portion of the mass.

Complete blood workup was ordered and all results returned within normal limits. Subsequently ordered magnetic resonance imaging (MRI) demonstrated a giant in size, well-defined intermuscular soft tissue mass homogeneously isointense with fat, next to the sciatic nerve (Figure 1). Interestingly, the mass showed and intra-pelvic extension advancing the greater sciatic foramen and passing through the infrapiriform aperture. It was clear that the mass had both intra and extra pelvic extension. Based on the above the presumptive diagnosis of a lipoma was reached and the patient consented to proceed to surgical removal of the mass.

**Figure 2:**
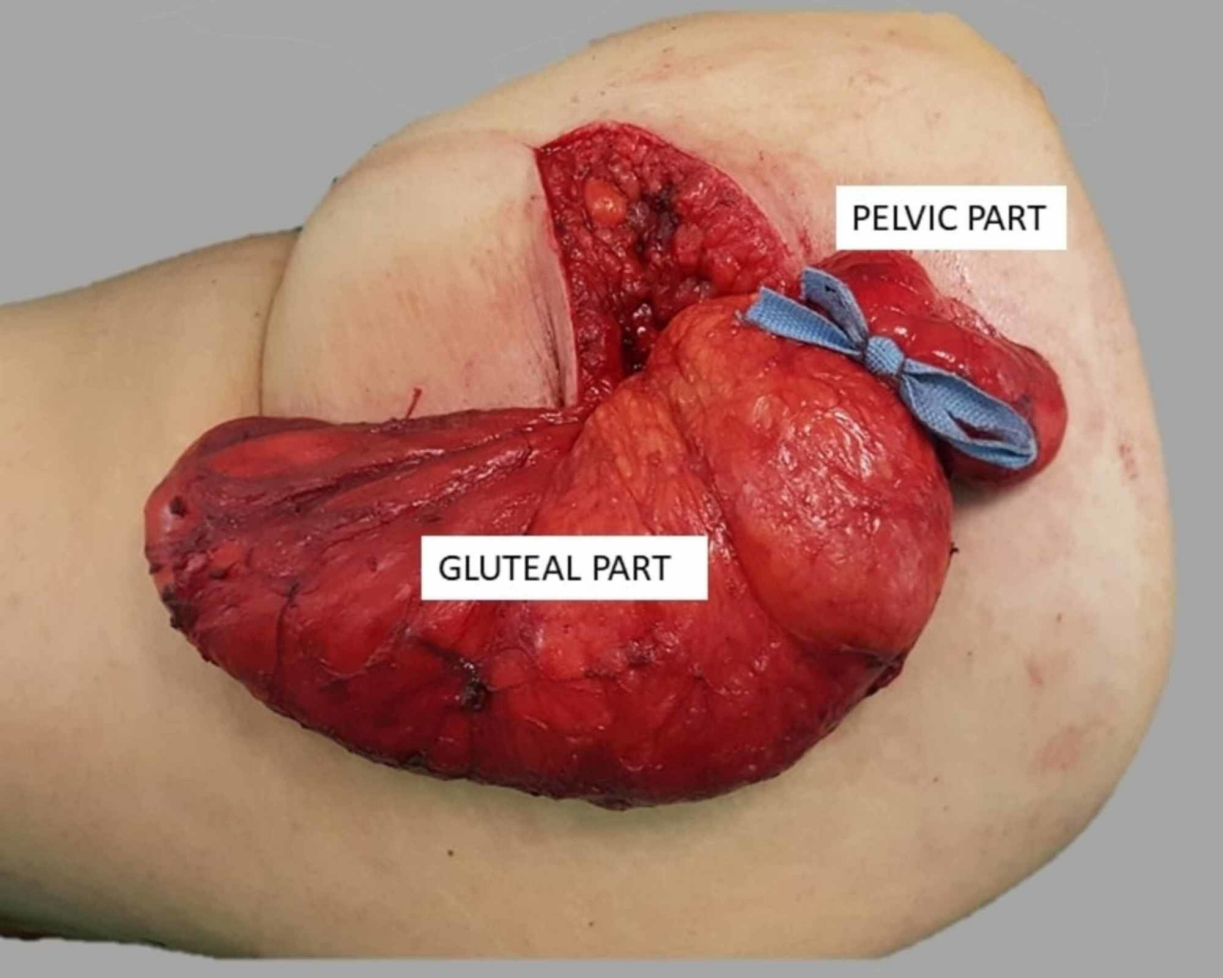
Intraoperative image of the tumor The part above the blue bow represents the pelvic portion of it (gluteal and pelvic part are indicated).

**Figure 3:**
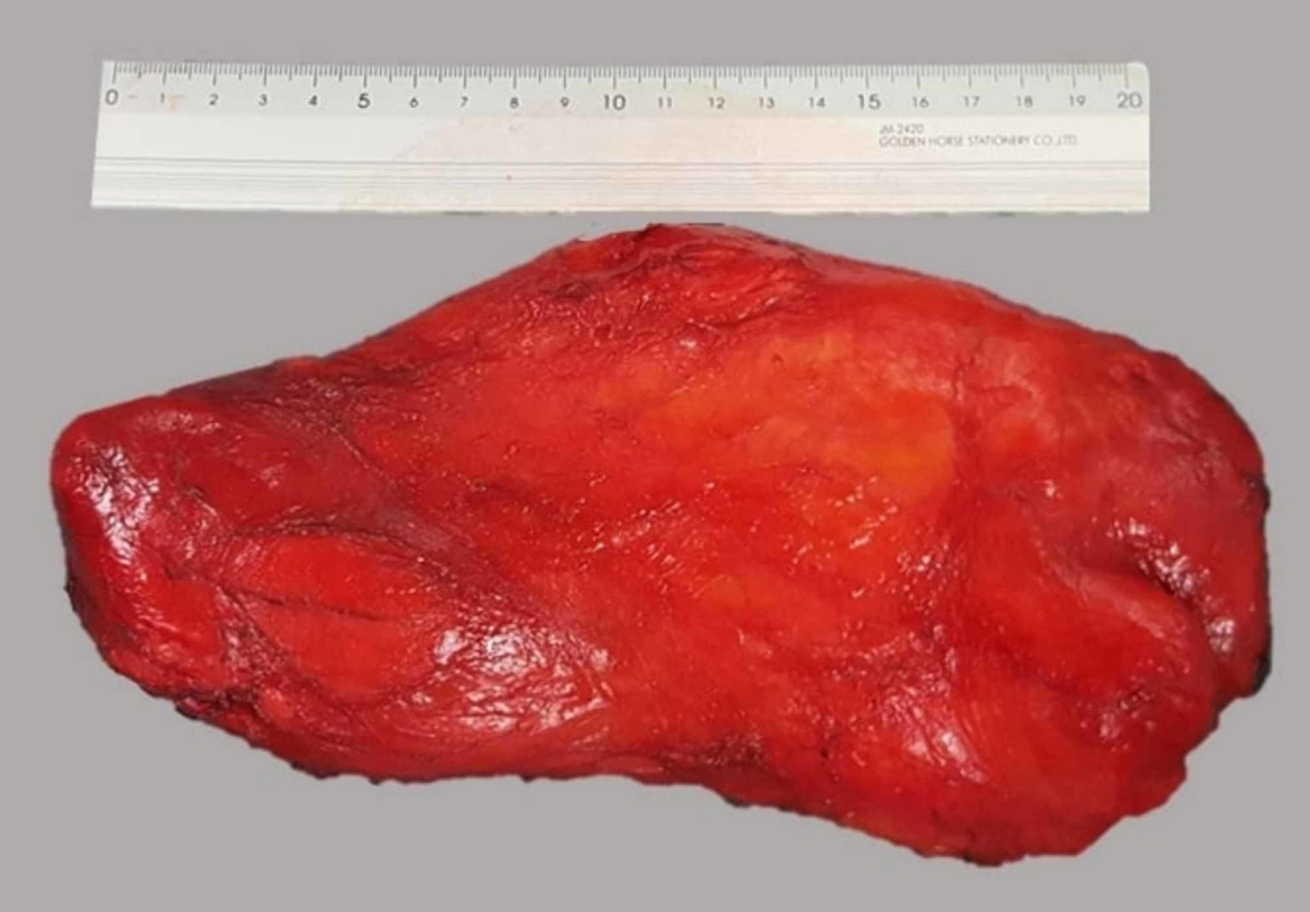
The excised specimen postoperatively

We decided to perform a wide transgluteal approach, thus avoiding a combined abdomino-perineal approach, because the lipoma was mainly located in the gluteus muscles, according to the imaging study. Incision was made in the skin, parallel to gluteus maximus fibers, along a line in between the major trochanter of the femur and the mid-sacrum, projecting the course of the piriformis muscle. The mass was carefully removed with meticulous blunt dissection and enucleation from the adjacent structures. The herniated, through the sciatic foramen, intra-pelvic portion of the lesion, was possible to withdraw and totally resected. Sciatic nerve and gluteal vessels were identified and left intact. The mass was entirely removed in an extracapsular plane thus ensuring a complete resection (Figure 2). The surgical field was thoroughly irrigated and hemostasis was easily achieved. The patient had an uneventful postoperative course and she was discharged three days later. Subsequently ordered pathological examination confirmed the diagnosis of a lobulated adipose tissue mass measuring 22 x 10.4 x 4.5 cm (Figure 3). Histopathological examination confirmed the diagnosis of lipoma. No signs of atypia or malignancy were observed. Two years postoperatively, the patient is recurrence free.

## Discussion

We reported on a rare case of an accidental ultrasound finding of a giant intermuscular gluteal lesion with intrapelvic extension, posing diagnostic and therapeutic dilemmas, since malignancy should be ruled out [5]. Sciatic hernia cases are extremely rare. Losanoff et al., in their literature review, reported that up to 99 cases have been published during the last century [6].

The exact etiology still remains uncertain, among other factors piriform muscle atrophy and neuromuscular disease have been also suggested as possible triggering factors, but intra-abdominal adhesions and elevated abdominal pressure should be also considered [7-8]. In the presented case no such associated factors were retrieved from the medical records of the patient.

Sciatic hernias may expand via either the lesser sciatic foramen or the supra- or the infra-piriform space. Our patient was symptom free, but other patients may complain of pain radiating over the buttock, thigh, back, even the pelvis [9]. Hernia’s content could be ovary and fallopian tube, ureter or bladder, small bowel and colon even omentum and Meckel’s diverticulum [6,9-11]. In the above-mentioned cases, respective symptoms include pelvic pain, ureteric colic and urinary tract infections, nausea and vomiting due to intestinal dilatation. Sciatic hernia may appear in adults and in children as well. Female predomination has been demonstrated the most likely due to larger pelvis and foramina in this gender [7,11]. Yu et al. reported on a small bowel obstruction due to incarcerated sciatic hernia containing a strangulated small bowel [12]. 

Preoperative diagnosis is mainly based on MRI and/or computed tomography (CT) scan since they depict size, extend and adjacent structures of the tumor. Depending on the presenting symptoms, other diagnostic modalities may include intravenous pyelography, plain X-ray, cystography, ultrasound [7]. Physical examination does not provide reliable information because of the tumor’s depth. Dulskas et al. reported on a case of a palpable gluteal lipoma, slightly reducible [2].

In our case, preoperative MRI delineated the lesion precisely, showing that it was an intermuscular lipoma, developed in between gluteal muscular fascicles, which favors complete resection of the lesion with digital dissection without resecting any muscles in order to achieve clear margins. Wide excision is regarded curative, since partial removal may lead to recurrence and further complications [7]. To the best of our knowledge, this is the sixth reported case of a lipoma herniating through the sciatic foramen [2]. Notably, Litchinko et al. published the excision of a 20 kg gluteal lipoma [13]

Transabdominal approach is strongly suggested for patients with bowel symptomatology such as incarceration or strangulation [6]. Skipworth et al. reported on a large sciatic hernia case quite similar to ours, with an intra- and extra-pelvic extension surgically removed through an abdomino-perineal approach [8]. An asynchronous abdomino-parasacral approach may be also used to resect giant lipomas [14]. An alternative technique has been suggested by transecting the sacrocpinous and sacrotuberous ligaments thus facilitating large intrapelvic tumors resection via a transgluteal approach [15]. In our case, the lipoma had a pelvic and a gluteal portion traversing the sciatic foramen. In lipomas, total resection is regarded curative and elective surgery for complete tumor resection was performed via a transgluteal approach only. The procedure should be performed with meticulous dissection in order to avoid intraoperative injury to the adjacent important structures such as the sciatic nerve and femoral cutaneous nerves, gluteal vessels and various abdominal organs such as the bowel, uterus and bladder. Prosthetic mesh or autologous free fascial flaps may be used during the procedure in certain cases of piriformis muscle atrophy [2]. The posterior approach to the hip was used, through the right gluteus maximus muscle, minimizing the length of the incision just to expose the piriformis muscle adequately. The incision extended along a line connecting the right major trochanter and the mid-sacrum, thus projecting the course of the right piriform muscle. Although this approach possesses increased risk of injury to the adjacent neurovascular structures, it facilitates the complete resection of the retroperitoneal portion of the tumor, traversing the greater sciatic foramen. Consequently, the combined abdominal-gluteal approach was avoided. Comparison of these two approaches cannot be done because of the lack of relevant studies. Singh et al. performed a robotically assisted laparoscopic repair of a sciatic hernia [16].

Right piriformis muscle was identified intact, without signs of atrophy or fibrosis and no patch was needed since sciatic defect was not found. Complete and uneventful surgical removal of the lesion was decided and achieved via a transgluteal approach, with careful digital dissection under constant visual control of both the pelvic and the gluteal portion of the mass. Surgeons involved in the treatment of such cases should always safely expose and preserve the sciatic and posterior femoral cutaneous nerve and assess the status of the piriformis muscle. The mass did not show any signs of infiltration during surgery and it was well delineated.

## Conclusions

A giant gluteal lipoma herniating through the sciatic foramen may accidentally be found during a pelvic ultrasound. Nowadays, MRI may suggest the diagnosis. Once the lesion is discovered, it should be completely resected. Surgeons involved in the treatment of such cases should keep in mind that an uneventful and safe surgical excision of a gluteal mass via a wide transgluteal approach still remains a reliable treatment choice.
